# Cumulative Serum Uric Acid and Its Time Course Are Associated With Risk of Myocardial Infarction and All‐Cause Mortality

**DOI:** 10.1161/JAHA.120.020180

**Published:** 2021-06-14

**Authors:** Xue Tian, Anxin Wang, Shouling Wu, Yingting Zuo, Shuohua Chen, Licheng Zhang, Dapeng Mo, Yanxia Luo

**Affiliations:** ^1^ Department of Epidemiology and Health Statistics School of Public Health Capital Medical University Beijing China; ^2^ Beijing Municipal Key Laboratory of Clinical Epidemiology Beijing China; ^3^ China National Clinical Research Center for Neurological Diseases Beijing Tiantan Hospital Capital Medical University Beijing China; ^4^ Department of Neurology Beijing Tiantan Hospital Capital Medical University Beijing China; ^5^ Department of Neurological Intervention Beijing Tiantan Hospital Capital Medical University Beijing China; ^6^ Department of Cardiology Kailuan Hospital North China University of Science and Technology Tangshan China

**Keywords:** all‐cause mortality, cumulative serum uric acid, early control, myocardial infarction, time course

## Abstract

**Background:**

Serum uric acid (SUA) has been demonstrated as a risk factor for myocardial infarction (MI) and all‐cause mortality; however, the impact of cumulative SUA (cumSUA) remains unclear. We aimed to investigate the association of cumSUA with MI risk and all‐cause mortality, and to further explore the effects of SUA accumulation time course.

**Methods and Results:**

The study enrolled 53 463 participants without a history of MI, and these participants underwent 3 examinations during 2006 to 2010. cumSUA from baseline to the third examination was calculated, multiplying mean values between consecutive examinations by time intervals between visits. Cox models estimated hazard ratios (HRs) and 95% CIs of MI and all‐cause mortality for cumSUA quartiles, hyperuricemia exposure duration, and SUA accumulation time course. During a median follow‐up of 7.04 years, 476 incident MIs and 2692 deaths occurred. In the fully adjusted model, a higher MI risk was observed in the highest cumSUA quartile (HR, 1.48; 95% CI, 1.10–1.99), in participants with longer hyperuricemia exposure duration (HR, 1.71; 95% CI, 1.06–2.73), and in participants with cumSUA≥median and a negative slope (HR, 1.58; 95% CI, 1.18–2.11). Similar associations persisted for all‐cause mortality.

**Conclusions:**

The risk of MI and all‐cause mortality increased with higher cumSUA and was affected by the SUA accumulation time course. Early SUA accumulation contributed more to MI risk and all‐cause mortality than later SUA accumulation with the same overall cumulative exposure, emphasizing the importance of optimal SUA control early in life.

**Nonstandard Abbreviations and Acronyms**
cumSUA: cumulative serum uric acid
SUA: serum uric acid

**Clinical Perspective**

**What Is New?**

- The risk of myocardial infarction and all‐cause mortality depends on long‐term cumulative exposure of serum uric acid (SUA), and the time course of SUA accumulation.
- The same cumulative SUA level acquired earlier in life resulted in a greater risk increased compared with high levels later in life.

**What Are the Clinical Implications?**

- Cumulative SUA is a practical and effective risk factor for myocardial infarction and all‐cause mortality in a large‐scale community population in China.
- These findings highlight the importance of optimal SUA control starting early in life.

Myocardial infarction (MI) is the leading cause of mortality from cardiovascular disease, and accounts for around 1 million deaths in China annually.^1^, ^2^ Thus, prevention of MI through greater understanding and reduction of risk factors has significant implications for public health and clinical practice. Serum uric acid (SUA), the end product of purine metabolism,^3^ has been proved to be associated with hypertension, diabetes mellitus, obesity, and dyslipidemia, all of which are principal contributors in the development and progression of MI and may reduce the longevity of the affected indivuduals.^4^, ^5^, ^6^, ^7^ However, whether SUA is an independent risk factor for MI and all‐cause mortality has been under debate.^8^, ^9^, ^10^, ^11^, ^12^, ^13^, ^14^ One major reason for these conflicting results may be attributed to the single measurement of SUA, which was not able to reflect the longitudinal variation and cumulative burden associated with elevated SUA levels.

Several recent studies have proposed the concept of cumulative exposure, a method that captures both the duration and intensity of a given parameter over several years, and has been used for the analysis of blood pressure, lipids, and hs‐CRP (high‐sensitivity C‐reactive protein) in several prospective cohort studies.^15^, ^16^, ^17^, ^18^, ^19^ However, few studies have evaluated the cumulative effect of SUA in relation to MI risk and all‐cause mortality.^20^, ^21^ Furthermore, it is still unclear whether the time course of cumulative SUA (cumSUA) accumulation is important in modulating the risk conferred by a given cumSUA level. Incorporating both the cumSUA level and its time course into a single risk parameter for future MI risk may provide additional information.

Therefore, in the present study, we aimed to (1) quantify the association of cumSUA with MI risk and all‐cause mortality; (2) evaluate the effect of hyperuricemia exposure duration; and (3) assess whether the time course of SUA accumulation affected the subsequent MI risk and all‐cause mortality.

## Methods

Data are available to researchers on request for purposes of reproducing the results or replicating the procedure by directly contacting the corresponding author.

### Study Population

**Figure 1:**
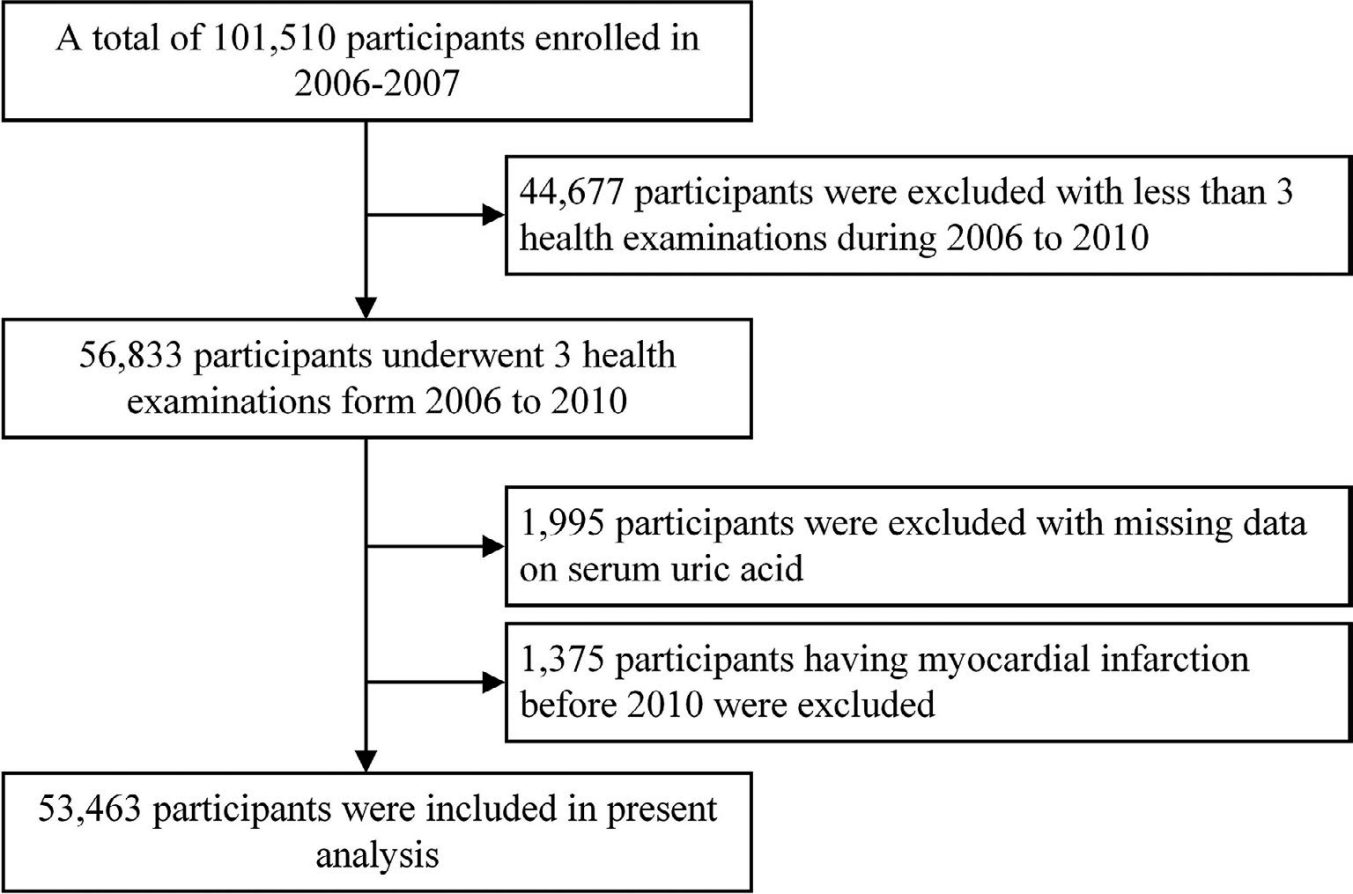
Flowchart of the study.

Data were derived from the Kailuan study, a prospective cohort study conducted in the Kailuan community in Tangshan City, China. Details of the study design have been described previously.^22^, ^23^ Briefly, from June 2006 to October 2007, 101 510 employees were recruited in the study (81 110 men and 20 400 women; aged 18–98 years) and received health examinations biennially until December 31, 2017 (Figure S1). We excluded 44 667 participants who did not have 3 times of health examinations between 2006 and 2010 and 1995 participants with missing SUA data. To minimize the possible effect of reverse causality, a further 1375 participants who had MI before 2010 were excluded. Consequently, 53 463 participants were included in the present analysis (Figure 1). A comparison of included and excluded participant characteristics is presented in Table S1. The study was performed according to the guidelines of the Declaration of Helsinki and was approved by the Ethics Committee of Kailuan General Hospital and Beijing Tiantan Hospital. All participants provided written informed consent.

### Calculation of cumSUA, Duration of Hyperuricemia Exposure, and Time Course of SUA Accumulation

Fasting blood samples were collected in the morning after an 8‐ to 12‐hour overnight fast and transfused into vacuum tubes containing EDTA. The concentration of SUA was examined with a commercial kit (Ke Hua Biological Engineering Corporation, Shanghai, China) using an automatic biochemical analyzer (Hitachi 7600, Tokyo, Japan), according to the manufacturer's instructions.

cumSUA was defined as the summed average SUA for each pair of consecutive examinations multiplied by the time interval between 2 consecutive examinations in years,^16^, ^18^, ^19^ and calculated as follows:

$$\text{CumSUA}=\left[\left({\text{SUA}}_{1}+{\text{SUA}}_{2}\right)/2\times {\text{time}}_{1‐2}\right]+\left[\left({\text{SUA}}_{2}+{\text{SUA}}_{3}\right)/2\times {\text{time}}_{2‐3}\right]$$

where SUA_1_, SUA_2_, and SUA_3_ indicate SUA at the baseline, second, and third examinations, and time_1–2_ and time_2–3_ indicate the participant‐specific time intervals between consecutive examinations in years (Figure S2). The means of time_1–2_ and time_2–3_ were 2.10 and 1.95 years, respectively.

Hyperuricemia was defined as an SUA level ≥420 μmol/L for men and ≥360 μmol/L for women.^24^ Hyperuricemia exposure duration was defined as the times of visits with hyperuricemia among the 3 visits, quantified as 0 years (never had hyperuricemia), 2 years (had hyperuricemia once), 4 years (had hyperuricemia twice), and 6 years (had hyperuricemia at all 3 study visits).^25^

Time course of SUA accumulation was categorized in 2 ways: a slope of SUA over time from 2006 to 2010 using the linear regression and the least‐squares principle, where SUA level was taken as the dependent variable, and time as the independent variable, with a positive or negative slope indicating an increase or decrease in SUA over time (Figure S2); or alternatively, the cumSUA between 2006 and 2008 and 2008 and 2010 were calculated as early and late SUA exposure measures, respectively.

### Assessment of Potential Covariates

Demographic and clinical characteristics, including age, sex, education, income, smoking status, alcohol use, physical activity, and medical history, were collected via self‐reported questionnaires. Educational attainment was categorized as illiteracy or primary school, middle school, and high school or above. Income level was categorized as <800 and ≥800 yuan. Smoking status and alcohol use were classified as never, former, or current, according to self‐reported information. Physical activity was classified as inactive activity (<80 minutes activity per week) and active activity (≥80 minutes activity per week). Weight and height were measured, and body mass index was calculated as weight (kg)/height (m)^2^. Systolic blood pressure and diastolic blood pressure were measured 3 times with the participants in the seated position using a mercury sphygmomanometer, and the average of 3 readings was used in the analyses. All blood samples were tested using a Hitachi 747 autoanalyzer (Hitachi, Tokyo, Japan) at the central laboratory of the Kailuan Hospital. Fasting blood glucose was measured using the hexokinase/glucose‐6‐phosphate dehydrogenase method. Serum creatinine was measured using the sarcosine oxidase assay method. Estimated glomerular filtration rate was calculated using the creatinine‐based Chronic Kidney Disease Epidemiological Collaboration (2009) equation.^26^ Plasma hs‐CRP was measured using a high‐sensitivity particle‐enhanced immunonephelometric assay. Hypertension was defined as any self‐reported hypertension or use of antihypertensive drug, or blood pressure ≥140/90 mm Hg. Diabetes mellitus was defined as any self‐reported diabetes mellitus or use of glucose‐lowering drugs, or fasting blood glucose ≥7 mmol/L. Dyslipidemia was defined as any self‐reported history or use of lipid‐lowering drugs, or serum total cholesterol ≥5.17 mmol/L, triglyceride ≥1.69 mmol/L, low‐density lipoprotein cholesterol ≥3.62 mmol/L, or high‐density lipoprotein cholesterol ≤1.04 mmol/L.

### Ascertainment of Outcomes

The primary outcomes of interest were incident MI and all‐cause mortality. The diagnosis of MI events was confirmed from biennial personal interviews, discharge summarized from the 11 hospitals, and medical records from medical insurance, using the *International Classification of Diseases, Tenth Revision*, code I21 for MI. Diagnosis of MI was based on a combination of chest pain symptoms, electrocardiographic signs, and cardiac enzyme levels.^27^ All‐cause mortality was defined as death from any cause and ascertained annually by professional physicians on the basis of examination of death certificates from provincial vital statistics offices.

### Statistical Analysis

Baseline characteristics are presented as mean (SD) for continuous variables or frequency (percentage) for categorical variables. Means and proportions between groups were compared using Student *t* test, ANOVA, or χ^2^ test, as appropriate. Person‐years was calculated from the date of the 2010 interview to the first occurrence of MI, mortality, or the end of the study, whichever came first. The incidence rate was calculated by dividing the number of incident cases by the total follow‐up duration (person‐years). The MI and all‐cause mortality probabilities were estimated by Kaplan‐Meier method and compared by log‐rank test.

Multivariable Cox proportional hazard regression models were used to assess the association of cumSUA, hyperuricemia exposure duration, and the time course of SUA accumulation with the MI risk and all‐cause mortality by calculating the hazard ratio (HR) and corresponding 95% CI. To adjust for potential confounding factors, 4 models were built systematically as follows: model 1 was unadjusted; model 2 was adjusted for age and sex; model 3 was further adjusted for education, income, smoking status, drinking status, physical activity, history of hypertension, diabetes mellitus, and dyslipidemia, body mass index, systolic blood pressure, diastolic blood pressure, and fasting blood glucose; and model 4 was further adjusted for antihypertensive agents, diuretics, hypoglycemic agents, lipid‐lowering agents, estimated glomerular filtration rate, and hs‐CRP. *P* values for trend were computed when categorical variables were used as ordinal variables. In addition, restricted cubic splines were used to examine the shape of the association between cumSUA and outcomes with 5 knots (at the 5th, 25th, 50th, 75th, and 95th percentiles). The reference point for cumSUA was the median (819.76 μmol/L×year) of the reference group (the first quartile), and the HR was adjusted for all confounding variables.

Additional analyses were performed to evaluate the robustness of the findings. First, when assessing the association of cumSUA exposure measures with MI risk, the Fine‐Gray competing risk models, considering non–cardiovascular disease deaths as competing risk events, were used as a sensitivity analysis. Second, considering other common diseases may have additional effects on all‐cause mortality, another sensitivity analysis was performed, excluding participants with cardiovascular or cerebrovascular disease during the follow‐up, when the association of cumSUA exposure measures with risk of all‐cause mortality was assessed. Finally, subgroup analyses were performed stratified by age (<60 versus ≥60 years), sex (women versus men), history of hypertension, diabetes mellitus, and dyslipidemia (no versus yes), body mass index (<25 versus ≥25 kg/m^2^), estimated glomerular filtration rate (<90 versus ≥90 mL/min per 1.73 m^2^), and hs‐CRP (<3 versus ≥3 mg/L). Interactions between subgroups were tested using likelihood ratio tests comparing models with and those without multiplicative interaction terms.

All analyses were conducted using SAS version 9.4 (SAS Institute Inc, Cary, NC). A 2‐sided *P*<0.05 was considered statistically significant.

## Results

### Baseline Characteristics

The characteristics of participants according to quartiles of cumSUA are presented in Table 1. The mean age of enrolled participants was 49.14±11.81 years, and 40 789 (76.29%) were men. Participants with higher cumSUA were more likely to be older, to be men, to be educated, to have higher income, to be more current smokers, to be current alcohol takers, to have active physical activity, to have higher prevalence of hypertension, diabetes mellitus, and dyslipidemia, to be more antihypertensive agent, diuretic, hypoglycemia agent, and lipid‐lowering agent users, and to have higher body mass index, systolic blood pressure, diastolic blood pressure, and hs‐CRP, but lower estimated glomerular filtration rate, levels, compared with participants in the lowest cumSUA quartile. When participants were categorized by cumSUA < or ≥ the median (1113.09 μmol/L×year) and a positive (or negative) slope of SUA, a similar trend of baseline characteristics was observed in participants with high cumSUA and a negative slope of SUA (Table S2).

**Table 1:** Baseline Characteristics of Participants, According to Quartiles of cumSUA

Characteristics	Overall	cumSUA, μmol/L×year				*P* Value
		Quartile 1 (<917.68)	Quartile 2 (917.69–1113.09)	Quartile 3 (1113.10–1357.41)	Quartile 4 (≥1357.42)	
No. of participants	53 463	13 365	13 365	13 365	13 365	
Age, y	49.14±11.81	45.77±10.32	48.5±11.36	50.47±11.82	51.83±12.73	<0.0001
Men, n (%)	40 789 (76.29)	8073 (60.40)	9747 (72.92)	10 960 (82.00)	12 009 (89.85)	<0.0001
High school or above, n (%)	4228 (8.10)	724 (5.52)	869 (6.65)	1107 (8.52)	1528 (11.72)	<0.0001
Income ≥800 yuan, n (%)	8093 (15.51)	1306 (9.96)	1749 (13.40)	2194 (16.89)	2844 (21.84)	<0.0001
Current smoker, n (%)	18 130 (34.73)	3077 (23.46)	4190 (32.10)	5082 (39.10)	5781 (44.37)	<0.0001
Current alcohol use, n (%)	20 714 (39.67)	3379 (25.75)	4567 (34.99)	5711 (43.92)	7057 (54.15)	<0.0001
Active physical activity, n (%)	7556 (14.13)	1097 (8.21)	1585 (11.86)	2208 (16.52)	2666 (19.95)	<0.0001
Hypertension, n (%)	5950 (11.13)	627 (4.69)	1079 (8.07)	1586 (11.87)	2658 (19.89)	<0.0001
Diabetes mellitus, n (%)	1452 (2.72)	282 (2.11)	361 (2.70)	412 (3.08)	397 (2.97)	<0.0001
Dyslipidemia, n (%)	3214 (6.01)	336 (2.51)	536 (4.01)	877 (6.56)	1465 (10.96)	<0.0001
Antihypertensive agents, n (%)	5167 (9.66)	512 (3.83)	885 (6.62)	1382 (10.34)	2388 (17.87)	<0.0001
Diuretics, n (%)	567 (1.09)	23 (0.18)	63 (0.48)	138 (1.06)	343 (2.63)	<0.0001
Hypoglycemic agents, n (%)	1120 (2.09)	223 (1.67)	275 (2.06)	321 (2.40)	301 (2.25)	0.0002
Lipid‐lowering agents, n (%)	482 (0.90)	49 (0.37)	75 (0.56)	128 (0.96)	230 (1.72)	<0.0001
Body mass index, kg/m^2^	25.07±3.48	24.40±3.45	24.76±3.43	25.20±3.44	25.93±3.41	<0.0001
Systolic blood pressure, mm Hg	128.59±20.03	125.61±19.14	127.54±19.34	129.09±20.01	132.13±21.00	<0.0001
Diastolic blood pressure, mm Hg	82.68±11.42	81.5±11.26	82.22±11.12	82.91±11.41	84.09±11.74	<0.0001
Fasting blood glucose, mmol/L	5.40±1.55	5.47±1.72	5.43±1.59	5.36±1.50	5.33±1.35	<0.0001
eGFR, mL/min per 1.73 m^2^	84.17±25.28	86.05±26.77	84.73±25.29	83.67±22.29	82.21±26.35	<0.0001
hs‐CRP, mg/L	2.35±6.54	1.84±5.51	2.21±7.48	2.63±7.10	2.72±5.83	<0.0001

Data are given as mean±SD unless otherwise indicated. cumSUA indicates cumulative serum uric acid; eGFR, estimated glomerular filtration rate; and hs‐CRP, high‐sensitivity C‐reactive protein.

### Association Between cumSUA and Risk of MI and All‐Cause Mortality

**Figure 2:**
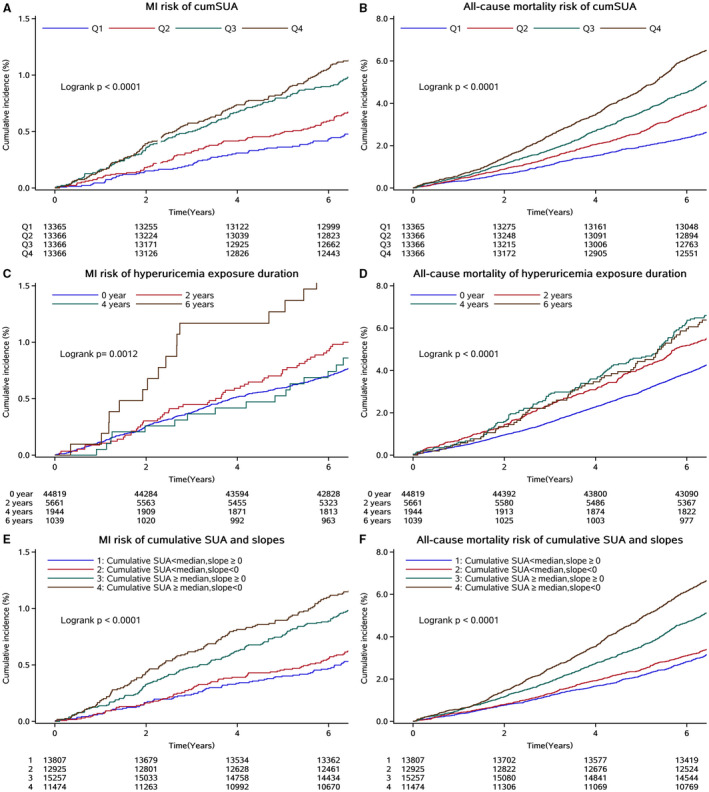
Kaplan‐Meier curve of myocardial infarction (MI) and all‐cause mortality incidence rate by quartiles (Qs) of cumulative serum uric acid (cumSUA) (**A** and **B**), cumulative exposure duration of hyperuricemia (**C** and **D**), and cumSUA and slope subgroups (**E** and **F**). SUA indicates serum uric acid.

During a median follow‐up of 7.04 years, 476 individuals (0.89%) developed MI and 2692 individuals (5.04%) died. The incidence rate of MI and all‐cause mortality was 1.31 (95% CI, 1.19–1.43) and 7.36 (95% CI, 7.08–7.64) per 1000 person‐years, respectively. The MI and all‐cause mortality rates, stratified by quartiles of cumSUA, are shown in Figure 2A and 2B. The data show that higher cumSUA exposure was associated with greater risk of MI and all‐cause mortality during the 7.04‐year follow‐up (*P*<0.0001 for log‐rank test). Higher incidence rate of MI and all‐cause mortality was also observed in participants with greater cumulative exposure time of hyperuricemia (*P*=0.0012 and *P*<0.0001, respectively; Figure 2C and 2D), and in the groups of participants with cumSUA levels ≥ median and a negative slope of SUA (*P*<0.0001; Figure 2E and 2F).

**Figure 3:**
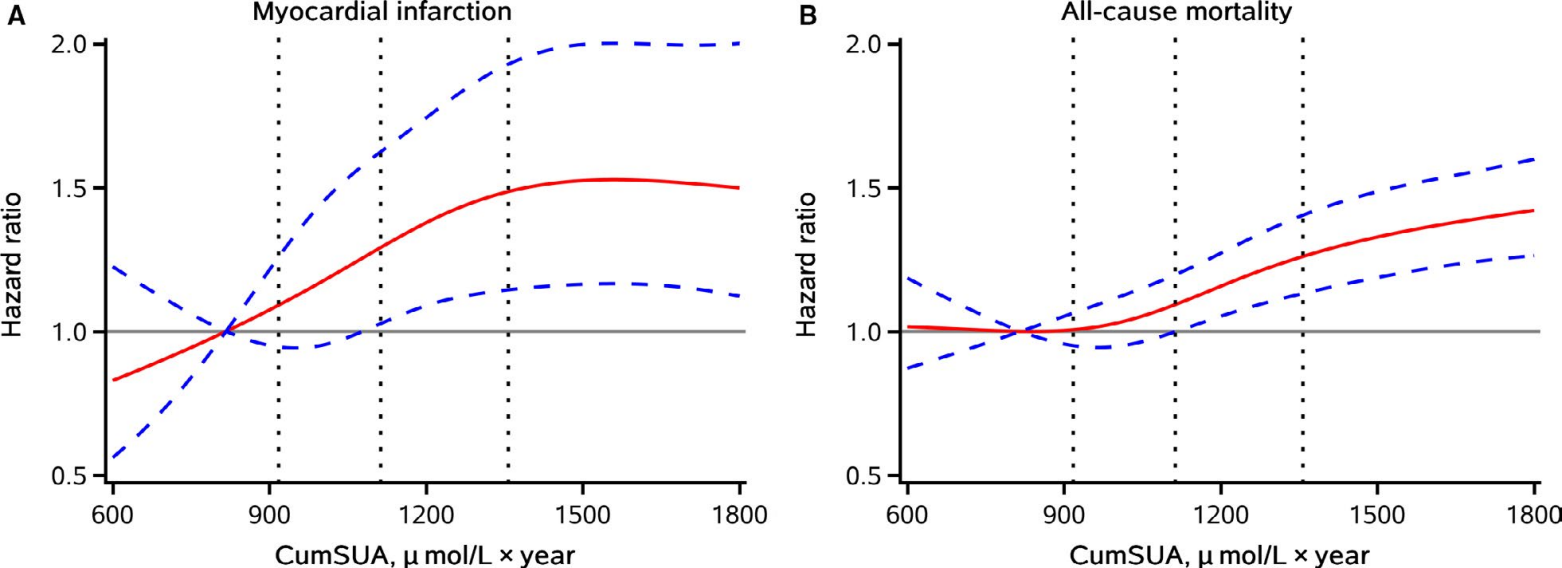
Hazard ratios (HRs) and 95% CIs for cumulative serum uric acid (cumSUA) with risk of myocardial infarction (**A**) and all‐cause mortality (**B**) by using restricted cubic spline regression with 4 knots placed at the 5th, 35th, 65th, and 95th percentiles of cumSUA. The red line represents HR, and blue lines represent 95% CI. Adjusted for age, sex, education, income, smoking status, drinking status, physical activity, history of hypertension, diabetes mellitus, and dyslipidemia, antihypertensive agents, diuretics, hypoglycemic agents, lipid‐lowering agents, body mass index, fasting blood glucose, systolic blood pressure, diastolic blood pressure, estimated glomerular filtration rate, and hs‐CRP (high‐sensitivity C‐reactive protein).

The associations of cumSUA exposure measures with MI risk and all‐cause mortality are presented in Tables 2 and 3. After adjustment for potential confounders, the risk of MI and all‐cause mortality was increased in the highest quartile compared with the lowest quartile of cumSUA, with HRs of 1.49 (95% CI, 1.11–2.00; *P* for trend=0.0032) for MI and 1.42 (95% CI, 1.26–1.61; *P* for trend<0.0001) for all‐cause mortality. Multivariable‐adjusted spline regression models showed J‐shaped associations between cumSUA and MI risk and all‐cause mortality (Figure 3A and 3B). Furthermore, when comparing the participants with the longest to the shortest cumulative duration of hyperuricemia exposure, participants with a 6‐year exposure duration of hyperuricemia had 1.72‐fold higher risk of MI (HR, 1.72; 95% CI, 1.07–2.76) and 1.39‐fold higher risk of all‐cause mortality (HR, 1.39; 95% CI, 1.10–1.77).

**Table 2:** HRs and 95% CIs for the Risk of MI, Stratified by cumSUA

Index	Case, n (%)	Incidence Rate*	Model 1	Model 2	Model 3	Model 4	Sensitivity Analysis^†^
cumSUA, μmol/L×year							
Quartile 1	73 (0.55)	0.78 (0.62–0.98)	Reference	Reference	Reference	Reference	Reference
Quartile 2	100 (0.75)	1.08 (0.89–1.32)	1.40 (1.04–1.90)	1.17 (0.86–1.59)	1.17 (0.86–1.58)	1.16 (0.86–1.57)	1.16 (0.85–1.58)
Quartile 3	141 (1.05)	1.56 (1.32–1.84)	2.02 (1.52–2.68)	1.50 (1.13–2.00)	1.48 (1.11–1.98)	1.47 (1.10–1.97)	1.46 (1.09–1.96)
Quartile 4	162 (1.21)	1.85 (1.58–2.15)	2.42 (1.84–3.19)	1.61 (1.21–2.14)	1.50 (1.11–2.01)	1.49 (1.11–2.00)	1.46 (1.08–1.98)
*P*_trend_			<0.0001	<0.0001	0.0027	0.0032	0.0047
Exposure duration of hyperuricemia, y^‡^							
0	376 (0.84)	1.23 (1.11–1.36)	Reference	Reference	Reference	Reference	Reference
2	60 (1.06)	1.58 (1.22–2.03)	1.29 (0.98–1.69)	1.21 (0.92–1.59)	1.10 (0.84–1.45)	1.10 (0.84–1.45)	1.09 (0.82–1.43)
4	21 (1.08)	1.62 (1.06–2.48)	1.33 (0.85–2.06)	1.20 (0.77–1.86)	1.06 (0.68–1.65)	1.05 (0.68–1.64)	1.02 (0.66–1.60)
6	19 (1.83)	2.75 (1.75–4.31)	2.25 (1.42–3.57)	2.01 (1.27–3.19)	1.76 (1.10–2.81)	1.72 (1.07–2.76)	1.67 (1.04–2.71)
*P*_trend_			<0.0001	0.0034	0.0549	0.0639	0.0955
Combination of cumSUA and SUA slope^§^							
cumSUA<median, slope≥0	78 (0.56)	0.81 (0.65–1.01)	Reference	Reference	Reference	Reference	Reference
cumSUA<median, slope<0	95 (0.74)	1.06 (0.86–1.29)	1.30 (0.96–1.76)	1.24 (0.92–1.67)	1.22 (0.90–1.64)	1.22 (0.90–1.65)	1.22 (0.90–1.65)
cumSUA≥median, slope≥0	160 (1.05)	1.57 (1.34–1.83)	1.95 (1.49–2.56)	1.53 (1.16–2.01)	1.45 (1.10–1.92)	1.45 (1.10–1.92)	1.44 (1.09–1.90)
cumSUA≥median, slope<0	143 (1.25)	1.88 (1.59–2.21)	2.34 (1.78–3.08)	1.67 (1.26–2.22)	1.59 (1.19–2.12)	1.58 (1.19–2.12)	1.56 (1.16–2.08)
*P*_trend_			<0.0001	<0.0001	<0.0001	0.0010	0.0014

Model 1: unadjusted. Model 2: adjusted for age and sex. Model 3: further adjusted for education, income, smoking status, drinking status, physical activity, history of hypertension, diabetes mellitus, and dyslipidemia, body mass index, systolic blood pressure, diastolic blood pressure, and fasting blood glucose. Model 4: further adjusted for antihypertensive agents, diuretics, hypoglycemic agents, lipid‐lowering agents, estimated glomerular filtration rate, and hs‐CRP (high‐sensitivity C‐reactive protein). cumSUA indicates cumulative SUA; HR, hazard ratio; MI, myocardial infarction; and SUA, serum uric acid.

* Incidence rate per 1000 person‐years.

^†^ Sensitivity analysis used competing risk model, considering death as a competing risk, and adjusted for covariables in model 4.

^‡^ Hyperuricemia was defined as SUA ≥420 μmol/L in men and ≥360 μmol/L in women.

^§^ Median of cumSUA was 1113.09 μmol/L×year.

**Table 3:** HRs and 95% CIs for the Risk of All‐Cause Mortality, Stratified by cumSUA and SUA Slope

Index	Case, n (%)	Incidence Rate*	Model 1	Model 2	Model 3	Model 4	Sensitivity Analysis^†^
cumSUA, μmol/L×year							
Quartile 1	415 (3.11)	4.41 (4.00–4.85)	Reference	Reference	Reference	Reference	Reference
Quartile 2	599 (4.48)	6.47 (5.97–7.01)	1.47 (1.30–1.67)	1.10 (0.97–1.24)	1.14 (1.01–1.30)	1.14 (1.00–1.29)	1.11 (0.97–1.27)
Quartile 3	744 (5.57)	8.18 (7.61–8.79)	1.87 (1.66–2.11)	1.16 (1.03–1.31)	1.27 (1.12–1.44)	1.26 (1.11–1.42)	1.20 (1.06–1.37)
Quartile 4	934 (6.99)	10.60 (9.93–11.30)	2.45 (2.18–2.75)	1.28 (1.14–1.45)	1.45 (1.28–1.64)	1.42 (1.26–1.61)	1.38 (1.21–1.57)
*P*_trend_			<0.0001	<0.0001	<0.0001	<0.0001	<0.0001
Exposure duration of hyperuricemia, y^‡^							
0	2139 (4.77)	6.95 (6.66–7.25)	Reference	Reference	Reference	Reference	Reference
2	341 (6.02)	8.92 (8.02–9.92)	1.29 (1.15–1.44)	1.16 (1.03–1.30)	1.18 (1.05–1.32)	1.17 (1.04–1.31)	1.14 (1.00–1.29)
4	139 (7.15)	10.70 (9.06–12.60)	1.55 (1.30–1.84)	1.32 (1.11–1.56)	1.35 (1.14–1.61)	1.33 (1.12–1.58)	1.34 (1.11–1.62)
6	73 (7.03)	10.50 (8.32–13.20)	1.51 (1.20–1.91)	1.30 (1.03–1.64)	1.43 (1.13–1.81)	1.39 (1.10–1.76)	1.34 (1.02–1.75)
*P*_trend_			<0.0001	<0.0001	<0.0001	<0.0001	0.0001
Combination of cumSUA and SUA slope^§^							
cumSUA<median, slope≥0	497 (3.60)	5.15 (4.72–5.63)	Reference	Reference	Reference	Reference	Reference
cumSUA<median, slope<0	517 (4.00)	5.73 (5.25–6.24)	1.11 (0.98–1.26)	1.08 (0.96–1.23)	1.09 (0.96–1.23)	1.09 (0.96–1.23)	1.08 (0.94–1.22)
cumSUA≥median, slope≥0	846 (5.54)	8.24 (7.71–8.82)	1.62 (1.45–1.81)	1.17 (1.05–1.31)	1.24 (1.11–1.39)	1.23 (1.10–1.38)	1.20 (1.06–1.35)
cumSUA≥median, slope<0	832 (7.25)	10.90 (10.20–11.60)	2.13 (1.91–2.38)	1.25 (1.11–1.40)	1.39 (1.23–1.56)	1.37 (1.22–1.54)	1.32 (1.16–1.50)
*P*_trend_			<0.0001	<0.0001	<0.0001	<0.0001	<0.0001

Model 1: unadjusted. Model 2: adjusted for age and sex. Model 3: further adjusted for education, income, smoking status, drinking status, physical activity, history of hypertension, diabetes mellitus, and dyslipidemia, body mass index, systolic blood pressure, diastolic blood pressure, and fasting blood glucose. Model 4: further adjusted for antihypertensive agents, diuretics, hypoglycemic agents, lipid‐lowering agents, estimated glomerular filtration rate, and hs‐CRP (high‐sensitivity C‐reactive protein). cumSUA indicates cumulative SUA; HR, hazard ratio; and SUA, serum uric acid.

* Incidence rate per 1000 person‐years.

^†^ Sensitivity analysis was adjusted for covariables in model 4 and further excluded those with cerebrovascular diseases before 2010.

^‡^ Hyperuricemia was defined as SUA ≥420 μmol/L in men and ≥360 μmol/L in women.

^§^ Median of cumSUA was 1113.09 μmol/L×year.

### Time Course of SUA Exposure and Risk of MI and All‐Cause Mortality

Results of association between time course of SUA exposure evaluated by adding the slope of SUA to the analysis or by splitting the overall cumSUA progression between 2006 and 2010 into an early (cumSUA between 2006 and 2008) and late accumulation (cumSUA between 2008 and 2010) and outcomes are presented in Table 4. After adjustment for covariates, participants with a negative slope of SUA time course tended to have a potential higher risk of MI (HR, 1.11; 95% CI, 0.93–1.33) and all‐cause mortality (HR, 1.09; 95% CI, 1.01–1.18). In accordance with this, results also showed that later cumSUA (cumSUA between 2008 and 2010) was not associated with risk of MI (*P*=0.4916) or all‐cause mortality (*P*=0.0801) after adjusting for early cumSUA (cumSUA between 2006 and 2008). When considering the combined effect of cumSUA and slope, we found individuals with cumSUA ≥ median and a negative slope of SUA had the highest risk of MI (HR, 1.58; 95% CI, 1.19–2.12; Table 2) and all‐cause mortality (HR, 1.37; 95% CI, 1.22–1.54; Table 3) among the 4 groups.

**Table 4:** Association of Time Course of SUA With Risk of MI and All‐Cause Mortality

Outcomes	Slope of SUA*			cumSUA06–08		cumSUA08–10^†^	
	<0	≥0	*P* Value	Per 100 μmol/L×year	*P* Value	Per 100 μmol/L×year	*P* Value
MI	1.11 (0.93–1.33)	Reference	0.1075	1.05 (1.01–1.10)	0.0120	1.02 (0.97–1.07)	0.4689
All‐cause mortality	1.09 (1.01–1.18)	Reference	0.0236	1.05 (1.04–1.07)	<0.0001	1.02 (0.99–1.04)	0.0895

Adjusted for age, sex, education, income, smoking status, drinking status, physical activity, history of hypertension, diabetes mellitus, and dyslipidemia, antihypertensive agents, diuretics, hypoglycemic agents, lipid‐lowering agents, body mass index, systolic blood pressure, diastolic blood pressure, fasting blood glucose, estimated glomerular filtration rate, and hs‐CRP (high‐sensitivity C‐reactive protein). cumSUA06–08 indicates cumulative SUA between 2006 and 2008; cumSUA08–10, cumulative SUA between 2008 and 2010; MI, myocardial infarction; and SUA serum uric acid.

* Further adjusted for cumulative SUA between 2006 and 2010.

^†^ Further adjusted for cumulative SUA between 2006 and 2008.

### Additional Analyses

Results of sensitivity analyses were consistent with the main analyses (Tables S3 and S4). In the subgroup analyses, the association between higher quartiles of cumSUA with risk of MI and all‐cause mortality was consistent and significant across subgroups generally (*P* for interaction >0.05 for all).

## Discussion

This study showed that the MI risk and all‐cause mortality depended on both cumSUA exposure and on the time course of SUA accumulation. Specifically, our study suggested that early high cumSUA contributed more to later risk of MI and all‐cause mortality when the same cumSUA was accumulated later in life. This highlights the importance of optimal SUA levels earlier in life, because reduced SUA later on, even when low enough to result in the same cumSUA at the same time point, does not fully reverse risk acquired by higher SUA levels earlier. Moreover, it is important to note that the results only suggest an apparent persistent increase in later MI risk and all‐cause mortality risk conferred by high SUA levels experienced early in life, but not indicating that there is no benefit in primary prevention lowering SUA, no matter when SUA lowering is started.

The relationship between SUA and MI has been debated with conflicting results in previous investigations. The AMORIS (Apolipoprotein Mortality Risk) study^9^ and the Rotterdam study^8^ have demonstrated a significant association between high SUA and MI. In contrast, the Tromsø Study^28^ and the NHANES (National Health and Nutrition Examination Survey) III study^29^ have failed to establish an independent association between SUA and MI. Furthermore, some studies have also shown that a significant relationship SUA and MI exists only in some specific populations, such as the nonhypertensive population.^30^ These conflicting results may be because previous studies have been reliant on a single time point measurement of SUA and were unable to examine the longitudinal association between long‐term SUA and MI. A single measurement of SUA is also subject to potential regression dilution bias and reverse causation issues. In addition, atherosclerosis, the major cause of MI, is a chronic progressive disease that begins early in life and develops over the course of decades before clinical manifestation.^15^ The cumulative exposure of SUA was related to both the intensity and duration of high SUA levels, and may reflect a more comprehensive effect of SUA on MI risk.

Similarly, with regard to SUA and all‐cause mortality, controversial results also exist. Some have suggested a J‐shaped, a U‐shaped, or a positive relationship,^10^, ^31^, ^32^ whereas others have suggested a reverse or null association.^11^, ^33^ Several studies have investigated the association between changes in SUA levels at 2 time points and subsequent risk of all‐cause mortality, with results showing that decreased SUA is associated with the risk of all‐cause mortality in patients with specific conditions, such as patients receiving maintenance hemodialysis or peritoneal dialysis.^33^, ^34^, ^35^ One study has assessed the relationship between long‐term changes in SUA and risk of mortality in the general population, with results showing that both decreased and increased SUA were associated with mortality.^20^ However, the time course of SUA changes was considered in these reports. In our study, we aimed to address these knowledge gaps and methodological limitations by calculating cumSUA with a long‐term follow‐up, allowing the time course of SUA accumulation to be assessed. We found that elevated cumSUA exposure and the time course of its accumulation were associated with risk of all‐cause mortality. Our finding showed a J‐shaped relationship between longitudinal cumSUA and all‐cause mortality, indicating the adverse effects of a longitudinal cumulative high SUA may be stronger than the accumulation of low SUA over time. Our study suggests that serial elevated levels of cumSUA may be more prognostic because it reflects less risk of misclassification.

It is noteworthy that the distribution of SUA levels differs by sex, and premenopausal women tend to have lower SUA levels than men because of the uricosuric effect of estrogens.^36^ Some studies have found that women showed a stronger relationship between SUA and MI or all‐cause mortality than men,^8^, ^9^ whereas some others have demonstrated a significant association was only observed in men,^32^, ^36^ or showed no significant sex difference in these associations at all.^31^, ^37^ In line with the latter reports, our subgroup analyses show that there is no significant interaction between sex and cumSUA in relation to the risk of MI and all‐cause mortality, indicating that elevated cumSUA levels have similar adverse effects on development of MI and all‐cause mortality in both sexes.

The association of cumSUA with MI risk and all‐cause mortality may be affected by administration of SUA‐lowering therapies. The CARES (Cardiovascular Safety of Febuxostat and Allopurinol in Patients With Gout and Cardiovascular Morbidities) trial has shown there was no significant difference between febuxostat and allopurinol with respect to rates of adverse cardiovascular disease events, but the risk of all‐cause mortality and cardiovascular mortality was higher in patients with febuxostat than with allopurinol. However, no control group was included in the trial, so the study was unable to compare the risk of all‐cause and cardiovascular mortality in subjects with and without uric acid–lowering treatment.^38^ Another meta‐analysis of 35 randomized controlled trials in patients with gout showed that SUA‐lowering therapy did not reduced the composite of cardiovascular disease death, nonfatal MI, nonfatal stroke, or all‐cause mortality compared with the placebo.^39^ Because the information on SUA‐lowering therapy was not available in our study, the question of whether cumSUA is a risk factor or simply a correlate (epiphenomenon) of cardiovascular risk clustered in subjects with elevated cumSUA level needs further investigations to answer.

The precise mechanisms underlying the positive association of high cumSUA with MI risk and all‐cause mortality remained rudimentary, but several possibilities have been proposed. First, SUA is a product of xanthine oxidoreductase, which is known to be one of the most important sources of oxygen reactive species. High cumSUA is therefore associated with increased vascular endothelial function, vascular smooth muscle cell proliferation, and oxidative stress, thereby increasing the risk of MI and all‐cause mortality.^40^, ^41^ Second, high cumSUA exerts a plethora of deleterious effects in cells and thus may be directly involved in the pathophysiological characteristics of MI and all‐cause mortality.^42^ Third, high cumSUA is correlated with almost all known cardiovascular risk factors, such as metabolic syndrome^43^ and chronic kidney disease^7^; thus, a higher level of cumSUA may be seen as a correlation of cardiovascular risk or an epiphenomenon of coexisting cardiometabolic risk factor.

Our study has some strengths, including a large sample size and long follow‐up time; in addition, SUA was measured repeatedly, and we used the cumulative value to accurately capture the longitudinal exposure of SUA, and considered the time course of cumSUA accumulation, which confers additional information beyond a single measured SUA level. However, our study still has some limitations. First, a history of gout and the use of medications for hyperuricemia or gout were not recorded in our study, which may have a potential effect on MI and all‐cause mortality. Second, we did not collect information on specific causes of death. Although we performed sensitivity analyses by excluding those whose deaths were from cardiovascular and cerebrovascular diseases, residual confounding death factors cannot be completely excluded. Finally, because our study was observational, we cannot establish a causal relationship of cumSUA with MI and all‐cause mortality.

## Conclusions

The risk of MI and all‐cause mortality depends on long‐term cumulative exposure of SUA, and the time course of SUA accumulation. The same cumSUA level acquired earlier in life resulted in a greater risk increased compared with high levels later in life. These findings highlight the importance of optimal SUA control starting early in life.

## Sources of Funding

This work was supported by the National Key Research and Development Program of China (No. 2018YFC1312800, 2018YFC1312801, 2018YFC1312400 and 2018YFC1312402), National Natural Science Foundation of China (grant No. 81773512), Beijing Municipal Administration of Hospitals Incubating Program (PX2020021), Beijing Excellent Talents Training Program (2018000021469G234), and Young Elite Scientists Sponsorship Program by China Association for Science and Technology (CAST) (2018QNRC001).

## Disclosures

None.

## Supporting information

Tables S1–S4
Figures S1–S2
