# Supplementary material for: Cumulative Serum Uric Acid and Its Time Course Are Associated With Risk of Myocardial Infarction and All‐Cause Mortality
Source: J Am Heart Assoc. 2021 Jun 14;10(13):e020180. doi: 10.1161/JAHA.120.020180 (PMC8403320; doi:10.1161/JAHA.120.020180)
Supplement: Supplementary file 1 — Tables S1–S4 Figures S1–S2 [file JAH3-10-e020180-s001.pdf]

SUPPLEMENTAL MATERIAL

Table S1. Baseline characteristics of excluded and included participants.

Characteristic	Excluded	Included	*P* value
No. of participants	48047	53463	
Age, years	55.04±12.86	49.14±11.81	<0.0001
Male, n (%)	40321 (83.92)	40789 (76.29)	<0.0001
High school or above, n (%)	2542 (5.61)	4228 (8.10)	<0.0001
Income≥1000RMB, n (%)	5922 (13.08)	8093 (15.51)	<0.0001
Current smoker, n (%)	15665 (33.98)	18130 (34.73)	0.0134
Current alcohol, n (%)	15938 (34.56)	20714 (39.67)	<0.0001
Active physical activity, n (%)	7725 (16.08)	7556 (14.13)	<0.0001
Hypertension, n (%)	7054 (14.68)	5950 (11.13)	<0.0001
Diabetes mellitus, n (%)	1798 (3.74)	1452 (2.72)	<0.0001
Dyslipidemia, n (%)	3046 (6.34)	3214 (6.01)	0.0300
Antihypertensive agents, n (%)	6147 (12.79)	5167 (9.66)	<0.0001
Diuretics, n (%) Hypoglycemic	611 (1.35%)	567 (1.09%)	0.0002
agents, n (%) Lipid-lowering	1361 (2.83)	1120 (2.10)	<0.0001
agents, n (%) Body mass	481 (1.00)	482 (0.90)	0.1023
index, kg/m^2	25.01±3.51	25.07±3.48	0.0045
Systolic blood pressure, mmHg	133.87±21.87	128.59±20.03	<0.0001
Diastolic blood pressure, mmHg	84.40±12.12	82.68±11.42	<0.0001
Fasting blood glucose, mmol/L	5.57±1.83	5.40±1.55	<0.0001

eGFR, mL/min/1.73m^2	79.35±25.82	84.17±25.28	<0.0001
hs-CRP, mg/L	2.48±6.38	2.35±6.54	0.0015

eGFR, estimated glomerular filtration rate; hs-CRP, high-sensitivity C-reactive protein.

Table S2. Baseline characteristics of participants stratified by cumulative SUA and SUA slope.

Characteristics	CumSUA < median, slope ≥ 0	CumSUA < median, slope < 0	CumSUA ≥ median, slope ≥ 0	CumSUA ≥ median, slope < 0	*P* value
No. of participants (%)	13807(25.83)	12925(24.18)	15257(28.54)	11474(21.46)	
Age, years	47.40±10.90	46.86±10.97	49.57±12.37	53.24±11.90	<0.0001
Male, n (%)	8619 (62.42)	9202 (71.20)	13249 (86.84)	9719 (84.70)	<0.0001
High school or above, n (%)	870 (6.30)	723 (5.59)	1525 (10.00)	1110 (9.67)	<0.0001
Income≥1000RMB, n (%)	1556 (11.27)	1499 (11.60)	2770 (18.16)	2268 (19.77)	<0.0001
Current smoker, n (%)	3428 (24.83)	3839 (29.70)	6256 (41.00)	4607 (40.15)	<0.0001
Current alcohol, n (%)	3809 (27.59)	4137 (32.01)	7331 (48.05)	5437 (47.39)	<0.0001
Active physical activity, n (%)	1376 (9.97)	1306 (10.10)	2457 (16.10)	2417 (21.07)	<0.0001
Hypertension, n (%)	857 (6.21)	849 (6.57)	2128 (13.95)	2116 (18.44)	<0.0001
Diabetes mellitus, n (%)	319 (2.31)	324 (2.51)	395 (2.59)	414 (3.61)	<0.0001

Dyslipidemia, n (%)	483 (3.50)	389 (3.01)	1165 (7.64)	1177 (10.26)	<0.0001
Antihypertensive agents, n (%)	712 (5.16)	685 (5.30) 33	1886 (12.36)	1884 (16.42)	<0.0001
Diuretics, n (%) Hypoglycemic	53 (0.40)	(0.26) 247	225 (1.53)	256 (2.25)	<0.0001
agents, n (%) Lipid-lowering	251 (1.82)	(1.91) 55	304 (1.99)	318 (2.77)	<0.0001
agents, n (%) Body mass	69 (0.50)	(0.43) 24.70	184 (1.21)	174 (1.52)	<0.0001
index, kg/m^2	24.47±3.42	±3.44 127.15	25.52±3.40	25.62±3.48	<0.0001
Systolic blood pressure, mmHg	126.07±19.48	±18.94 82.31	130.03±20.67	131.38±20.32	<0.0001
Diastolic blood pressure, mmHg	81.46±11.25	±11.08 5.49	83.27±11.70	83.81±11.39	<0.0001
Fasting blood glucose, mmol/L	5.41±1.72	±1.59 85.11	5.33±1.44	5.36±1.42	<0.0001
eGFR, $mL/min/1.73m^2$	85.65±24.62	±27.45 1.66	84.27±26.1	81.17±21.81	<0.0001
hs-CRP, mg/L	2.37±8.21	±4.04	2.83±6.09	2.47±6.94	<0.0001
SUA slope, µmol/L/year	13.67±11.26	-14.51±12.62	17.85±15.36	-15.67±15.08	<0.0001

eGFR, estimated glomerular filtration rate; hs-CRP, high-sensitivity C-reactive protein.

Table S3. Subgroup analysis for the association between cumulative SUA and risk of myocardial infarction.

Variables	Cumulative serum uric acid, μmol/L × year				$P_{interaction}$
	Q1 (<917.68)	Q2 (917.69-1113.09)	Q3 (1113.10-1357.41)	Q4(≥1357.42)	
Age, years					
<60	Reference	1.39(0.98-1.98)	1.65(1.18-2.33)	1.55(1.09-2.21)	0.1183
≥60	Reference	0.65(0.35-1.19)	0.98(0.57-1.69)	1.10(0.65-1.88)	
Sex					
Female	Reference	1.04(0.32-3.39)	2.60(1.06-6.39)	3.85(1.44-10.28)	0.1005
Male	Reference	1.02(0.73-1.40)	1.43(1.06-1.93)	1.33(0.98-1.81)	
Hypertension					
No	Reference	1.11(0.80-1.55)	1.48(1.08-2.03)	1.47(1.06-2.03)	0.8092
Yes	Reference	1.23(0.54-2.80)	1.25(0.56-2.76)	1.38(0.64-2.99)	
Diabetes					

No	Reference	1.24(0.91-1.70)	1.57(1.16-2.13)	1.63(1.20-2.22)	0.1388
Yes	Reference	0.45(0.14-1.48)	0.69(0.24-2.01)	0.36(0.10-1.23)	
Dyslipidemia					
No	Reference	1.20(0.87-1.64)	1.48(1.09-2.00)	1.64(1.21-2.24)	0.1841
Yes	Reference	0.59(0.19-1.86)	1.05(0.38-2.87)	0.52(0.19-1.48)	
BMI, kg/m^2					
<25	Reference	1.14(0.72-1.78)	1.42(0.91-2.21)	1.48(0.94-2.34)	0.9551
≥25	Reference	1.18(0.78-1.79)	1.52(1.03-2.25)	1.55(1.05-2.28)	
eGFR, $mL/min/1.73m^2$					
<90	Reference	1.27(0.88-1.84)	1.35(0.94-1.93)	1.42(0.99-2.04)	0.3171
≥90	Reference	0.85(0.49-1.49)	1.66(1.01-2.74)	1.53(0.89-2.62)	
hs-CRP, mg/L					
<3	Reference	1.17(0.82-1.66)	1.32(0.94-1.86)	1.41(0.99-1.99)	0.7154

≥3	Reference	1.11(0.60-2.05)	1.71(0.98-3.00)	1.60(0.90-2.82)

BMI, body mass index; eGFR, estimated glomerular filtration rate; hs-CRP, high-sensitivity C-reactive protein

Adjusted for age, sex, history of hypertension, diabetes and dyslipidemia, antihypertensive agents, diuretics, hypoglycemic agents, lipid-lowering agents, body mass index, systolic blood pressure, diastolic blood pressure, fasting blood glucose, estimated glomerular filtration rate, and high-sensitivity C-reactive protein other than variable for stratification.

Table S4. Subgroup analysis for the association between cumulative SUA and risk of all-cause mortality.

Variables	Cumulative serum uric acid, μmol/L × year				$P_{interaction}$
	Q1 (<917.68)	Q2 (917.69-1113.09)	Q3 (1113.10-1357.41)	Q4(≥1357.42)	
Age, years					
<60	Reference	1.20(1.02-1.42)	1.30(1.10-1.54)	1.45(1.22-1.72)	0.4873
≥60	Reference	1.03(0.85-1.25)	1.15(0.96-1.39)	1.31(1.09-1.58)	
Sex					
Female	Reference	1.11(0.81-1.54)	1.21(0.85-1.72)	1.46(1.01-2.12)	0.8234
Male	Reference	1.13(0.98-1.29)	1.25(1.09-1.42)	1.40(1.23-1.60)	
Hypertension					
No	Reference	1.11(0.97-1.27)	1.22(1.07-1.39)	1.36(1.19-1.56)	0.7581
Yes	Reference	1.27(0.86-1.87)	1.39(0.96-2.01)	1.61(1.12-2.31)	
Diabetes					

No	Reference	1.13(0.99-1.29)	1.27(1.11-1.44)	1.42(1.25-1.62)	0.4133
Yes	Reference	1.22(0.79-1.88)	1.05(0.68-1.62)	1.21(0.77-1.88)	
Dyslipidemia					
No	Reference	1.14(1.00-1.30)	1.24(1.09-1.41)	1.43(1.26-1.63)	0.6605
Yes	Reference	0.98(0.55-1.75)	1.31(0.77-2.22)	1.21(0.72-2.04)	
BMI, kg/m^2					
<25	Reference	1.13(0.96-1.34)	1.36(1.15-1.60)	1.38(1.16-1.64)	0.1362
≥25	Reference	1.13(0.93-1.36)	1.12(0.93-1.35)	1.41(1.18-1.68)	
eGFR, mL/min/1.73m^2					
<90	Reference	1.12(0.97-1.31)	1.26(1.08-1.45)	1.39(1.20-1.61)	0.8610
≥90	Reference	1.16(0.91-1.46)	1.21(0.95-1.53)	1.46(1.14-1.86)	
hs-CRP, mg/L					
<3	Reference	1.16(1.00-1.34)	1.25(1.08-1.44)	1.37(1.18-1.59)	0.7788

≥3	Reference	1.04(0.82-1.34)	1.18(0.93-1.49)	1.39(1.10-1.75)

BMI, body mass index; eGFR, estimated glomerular filtration rate; hs-CRP, high-sensitivity C-reactive protein

Adjusted for age, sex, history of hypertension, diabetes and dyslipidemia, antihypertensive agents, diuretics, hypoglycemic agents, lipid-lowering agents, body mass index, systolic blood pressure, diastolic blood pressure, fasting blood glucose, estimated glomerular filtration rate, and high-sensitivity C-reactive protein other than variable for stratification.

Figure S1. Timeline of the study.

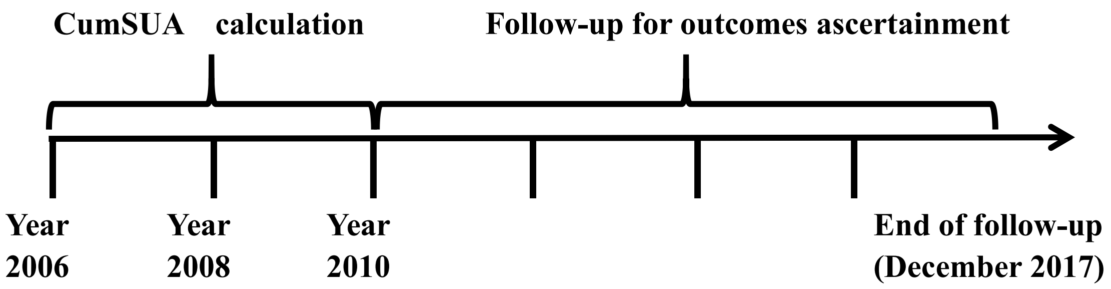

cumSUA, cumulative serum uric acid

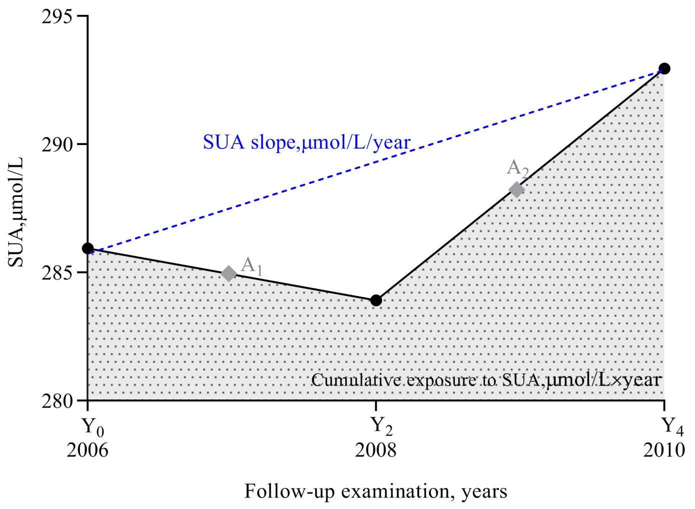

Figure S2. Cumulative SUA and SUA slope calculated across 3 examinations in 1 participants.

Average SUA between consecutive examinations as A1 and A2. Cumulative SUA was calculated as (A1 $\times$ $time_{06\text{-}08}$+A2 $\times$ $time_{08\text{-}10}$), showed by the dotted area, μmol/L × year.

SUA slope was obtained using a linear regression, SUA values at follow-up visits were used to calculate the ΔSUA.

Abbreviation: SUA, serum uric acid.
